# Cognition, prior aggression, and psychopathic traits in relation to impaired multimodal emotion recognition in psychotic spectrum disorders

**DOI:** 10.3389/fpsyt.2023.1111896

**Published:** 2023-06-22

**Authors:** Lennart Högman, Gabriela Gavalova, Petri Laukka, Marianne Kristiansson, Malin V. Källman, Hakan Fischer, Anette G. M. Johansson

**Affiliations:** ^1^Department of Psychology, Stockholm University, Stockholm, Sweden; ^2^Aleris Psychiatry, Täby Psychotic Disorders Clinic, Stockholm, Sweden; ^3^Department of Clinical Neuroscience, Karolinska Institute, Stockholm, Sweden; ^4^Centre for Psychiatry Research, Stockholm, Sweden; ^5^Swedish National Board of Forensic Medicine, Stockholm, Sweden

**Keywords:** emotion recognition, psychosis, schizophrenia, aggression, violence, psychopathy

## Abstract

**Background:**

Psychopathic traits have been associated with impaired emotion recognition in criminal, clinical and community samples. A recent study however, suggested that cognitive impairment reduced the relationship between psychopathy and emotion recognition. We therefore investigated if reasoning ability and psychomotor speed were impacting emotion recognition in individuals with psychotic spectrum disorders (PSD) with and without a history of aggression, as well as in healthy individuals, more than self-rated psychopathy ratings on the Triarchic Psychopathy Measure (TriPM).

**Methods:**

Eighty individuals with PSD (schizophrenia, schizoaffective disorder, delusional disorder, other psychoses, psychotic bipolar disorder) and documented history of aggression (PSD+Agg) were compared with 54 individuals with PSD without prior aggression (PSD-Agg) and with 86 healthy individuals on the Emotion Recognition Assessment in Multiple Modalities (ERAM test). Individuals were psychiatrically stable and in remission from possible substance use disorders. Scaled scores on matrix reasoning, averages of dominant hand psychomotor speed and self-rated TriPM scores were obtained.

**Results:**

Associations existed between low reasoning ability, low psychomotor speed, patient status and prior aggression with total accuracy on the ERAM test. PSD groups performed worse than the healthy group. Whole group correlations between total and subscale scores of TriPM to ERAM were found, but no associations with TriPM scores within each group or in general linear models when accounting for reasoning ability, psychomotor speed, understanding of emotion words and prior aggression.

**Conclusion:**

Self-rated psychopathy was not independently linked to emotion recognition in PSD groups when considering prior aggression, patient status, reasoning ability, psychomotor speed and emotion word understanding.

## 1. Introduction

The ability to correctly perceive and interpret others’ emotions is a critical part of living a full and inclusive community life. Emotion recognition is defined as the ability to correctly identify an emotion expressed by another person in their faces, gestures, posture as well as voice (content, prosody, tone, and forcefulness) (1). It is well established that individuals with schizophrenia have impairments in perceiving emotions in standardized Ekman faces either in briefly masked stimuli, degraded facial affect paradigms or static faces [see for example (2–5)].

Studies have suggested that severe negative symptoms in psychosis (6) and greater illness severity (7) are associated with greater impairment in emotion recognition. Deficits do not appear to be associated with a higher polygenic risk score for schizophrenia, even though 1st degree relatives exhibit similar impairments (8), suggesting a separate genetic or familial pathway of social cognition impairments. The deficits are stable during the course of the illness (9), yet mild or not present in the prodromal phase, when IQ is controlled for (10). This contrasts with other, stable, social cognitive deficits in schizophrenia, suggesting an interaction between emotion recognition and the development of positive symptoms of psychosis.

While findings on facial emotion recognition are robust in psychosis, it is less certain if these findings translate to impairments in interpreting others’ emotions in more ecologically valid test paradigms of body posture and motion. Studies in schizophrenia have tended to use short facial videos, ignoring body posture as a communicator of emotion (9, 11, 12). Where body posture has been used, it has been in the form of point-light displays, where a recent meta-analysis confirmed moderate to large impairments (13). Although there is a suggestion that it is primarily visual processing that is impaired in schizophrenia, and not specifically the reading of emotion into motion (13), higher point-light emotion recognition scores did predict having had prior relationships and it was found to be a significant contributor to psychosocial function, over and above neurocognitive function (14). Studies examining emotion perception in auditory paradigms (15) have found impaired preattentive processing of tonal information (15, 16) as well as stable impairments in ability during remitted phases (17). The impairments were associated with greater negative symptoms of schizophrenia (18). As to examining both visual and auditory emotion recognition, we have only found one small study examining visual (V), auditory (A) and auditory plus visual (A+V) stimuli suggesting impairments in V and A conditions but not when both were presented together (19).

Psychotic spectrum disorders (PSD), as opposed to the concept of schizophrenia spectrum disorders, can include bipolar disorder with psychotic symptoms, One study has shown that individuals with bipolar disorder exhibit less emotion recognition deficits than those with schizophrenia (20), less deficits but with significant overlap with schizophrenia if psychotic features (21), or equivalent performance when taking cognitive deficits into account (2). Another study still found differences between groups when accounting for cognitive ability (22), but this study did not differentiate between those with or without psychotic features. In other words, there appears to be some support for more severe emotion recognition impairment in those with psychotic features or more severe cognitive deficits.

If the ability to detect and decode emotional communications of facial expression, voice signals and body language is impaired then it is hypothesized that violence or other transgressions may occur in interpersonal situations. In fact, one of the models of socialized behavior, the violence inhibition mechanism, posits that sad facial affects (distress cues) function as a human submission response, which acts as a stop signal for aggressive behavior (23). So far, results point to emotion recognition, processing speed and education being the strong discriminators between individuals with psychosis who have been aggressive in forensic services to compared with those who have not been aggressive (24). Other major predisposing factors for aggression are drug use, younger male gender, a history of involuntary treatment, prior offending, as well as a longer duration of untreated psychosis (25), lower IQ and prior offending (26). Anger in others was the only emotion which individuals in the group exhibiting aggressive behavior were better able to identify (24), even though a further two studies found impairment also in anger recognition (27, 28). One study found that impaired recognition of sadness was linked to victimization prior to aggression, which in turn was linked with higher psychopathy ratings in a first episode of psychosis (29), which begs the question of how these are developmentally related.

As to PSD, variable findings have been found regarding the relationship of psychopathic traits to emotion recognition, perhaps associated with the low sample sizes in studies. Fullam and Dolan found similar discrepancies in emotion recognition between high and low psychopathy individuals in schizophrenia (30) as in the offender population (31). Individuals with antisocial personality disorder and schizophrenia showed impairment in recognizing fear faces (32), deficits over and above those of just having schizophrenia (11). Studies of emotion recognition in offenders with psychopathic traits have identified impairment across the six basic emotions (33), yet the link between psychopathic traits in offenders and emotion recognition was in one study found to be tempered by cognitive impairments (34). There may be a severity threshold effect of psychopathic traits on emotion recognition as utilizing the Triarchic psychopathy measure in undergraduate students did not yield a relationship between psychopathic traits and emotion recognition (35), nor was there a relationship between psychopathic traits and emotion recognition in employed individuals (36).

Emotion recognition research has mainly examined the six basic emotions of fear, sadness, anger, disgust, surprise, and happiness in facial emotion recognition; it is unclear if emotion recognition is worse in individuals with PSD with respect to a history of aggression if more ecologically valid multimodal emotion portrayals are used. Given the associations between cognitive function and emotion recognition in bipolar disorder (2, 22) and in PSD (37) it is paramount to take cognition into account to ascertain if psychopathic traits and/or a history of aggression is associated with greater impairments in emotion recognition in the PSD group. Furthermore, it is necessary to control for possible effects of impaired semantic understanding of emotion words and possible psychomotor slowing from antipsychotic medications. By combining these measures, we wish to test the hypotheses that (1) self-rated psychopathic traits will be higher in individuals with psychotic spectrum disorder with prior aggression (PSD+Agg) group, and this will be associated with lower accuracy of emotion recognition, (2) there will not be any differences in self-rated psychopathic traits between healthy individuals and individuals with PSD without a history of aggression (PSD-Agg) and (3) patient groups will have lower accuracy on emotion recognition than healthy individuals, the greatest impairment seen in those with PSD+Agg, Additionally, we hypothesize that self-rated psychopathy will be greatest in the PSD+Agg group, contributing to impairments in emotion recognition.

## 2. Methods

### 2.1. Participants

Individuals with psychotic spectrum disorders (PSD), here defined as schizophrenia, schizoaffective disorder, delusional disorder, other psychoses, bipolar disorder with psychotic features, were recruited as part of the Stockholm Forensic Psychiatric Project (SFPP). The study was established to investigate possible links between known epidemiological risk factors for aggression in those with psychotic spectrum disorders with psychological and biological functions. This sub-study is a cross-sectional cohort study of patients with the above-listed diagnoses who had been aggressive and receiving forensic psychiatric care, individuals with the same diagnoses but without a history of aggression toward others receiving care at general psychiatric outpatient facilities, and age and sex matched healthy individuals.

Inclusion criteria for individuals with PSD were age 20–60 years at time of consent; >3 months clinical stability at the time of consent and testing; no medication changes for the past 3 months; they may have had a history of substance abuse but be in remission for >3 months before testing was conducted. Individuals may have comorbid ADHD, personality disorders and mild intellectual disability. Sufficient Swedish to understand the study instructions and tasks was necessary for inclusion.

Exclusion criteria were brain trauma prior to psychosis onset, neurological disorders, and untreated endocrine disorders as well as moderate intellectual disability. Given that no individuals in PSD+Agg group had diabetes type 1, this was regarded as an exclusion criterion for remaining research subjects to exclude effects of small vessel disease.

PSD+Agg: Aggression is defined as an index offence of threatening behavior, assault, grievous bodily harm and manslaughter/murder. Individuals with index offences such as property theft, robbery, arson or deliberate fire setting were also recruited as long as observed and documented interpersonal aggression occurred prior to sentencing and was recorded in the case files from hospitals, and crime reports.

The recruitment took place at Forensic Psychiatry Stockholm, a medium secure treatment facility between 2015–2019. Excluding individuals with insufficient language skills (~35 individuals), those with gliotic brain lesions/documented brain trauma (~20 individuals), those living in supported accommodation >100 km outside of the Stockholm area (~50 individuals), as well as those with other diagnoses such as Huntington’s disease, dementia, moderate intellectual disability (~30 individuals) and individuals who did not meet inclusion criteria for period of stability >3 months or remission in drug abuse (~50 individuals), another approximately 35 individuals did not wish to receive information about the study. This left 94 individuals who were asked to participate in SFPP. Of these, 85 consented to the study of which three withdrew consent during the initial phase of participation and another two did not do the emotion recognition task, leaving 80 for this study.

PSD-Agg individuals were recruited from 2019 onwards at Psychiatry Northwest outpatient clinics (recruitment is ongoing). Each participant was paid 1,000 Swedish crowns before tax. Flyers at the clinic advertised the study, interested individuals contacted the study team. Additionally, individuals who treating staff identified as meeting inclusion criteria were approached and given information as well as the opportunity to say yes or no to the study. Participation rates were low: 15% of those informed about the study. Individuals did not have to provide a reason for non-participation, but those who did provide a reason mentioned full time work, not wanting to discuss the past, the effort involved in participation, and self-reported history of interpersonal aggression. Of 60 recruited individuals six were excluded because of active substance use as measured by alcohol use disorders identification test (AUDIT) (38), drug use disorders identification test (DUDIT) (39), blood Phosphatidylethanol (PEth) (40), insufficient Swedish or wrong diagnoses (severe PTSD with flashbacks that mimicked psychosis). A semi structured interview was performed with specific questions about previous threatening behavior, aggression in self-defense situations and unprovoked aggression. Prior aggression, ascertained at interview as well as mentions of previous death threats and physical aggression toward other people in the case record, resulted in the individual not being eligible to participate in the study.

Healthy individuals were recruited *via* the State Person Address Registry (SPAR) at the Swedish Tax Agency. The registry was contacted and given a list of sex and year of birth to match sex and age of recruited PSD persons. A request was made for three controls matching each year of birth and sex, living in selected postcode areas in the greater Stockholm County area. The Tax agency then performed a randomized computer selection based on these variables and supplied the designated researcher with a list of names and addresses who in turn sent out letters of invitation to these individuals asking them to contact the research team if they were interested in participating in the research. Interested individuals completed a short telephone interview designed to canvas exclusion criteria and, if they passed, they were booked at a mutually convenient time for research participation. These individuals were given 1,000 Swedish crowns before tax for lost work time. Repeated randomizations and letter requests occurred to obtain the required number of matched individuals, about 700 letters were sent to obtain 94 individuals of which eight were excluded from analyses because of high scores on AUDIT, DUDIT, or poor Swedish language skills.

### 2.2. Measures

#### 2.2.1. Individuals with psychotic spectrum disorder

Using case record review supplemented by semi-structured interviews, symptoms of psychiatric disorders were rated by one of two experienced psychiatrists or a resident in training according to psychotic and affective sections of WHO’s Schedule for Clinical Assessment in Neuropsychiatry 2.1 (41). Consensus ratings of symptoms and diagnoses were done between the raters. Type and extent of prior substance use was rated according to information in case record and interview. Current medication and dosages were recorded from chart review, doses of antipsychotic medication were converted according to Andreasen’s model to haloperidol equivalents per day (42). The number and types of aggressive acts were analyzed according to Cornell’s rating guide (43) based on (i) crime report from police for crimes person has been sentenced for, (ii) reports in the forensic psychiatric care assessment about previous crimes the person has been sentenced for, (iii) case records of observable aggressive incidents in hospital services prior to forensic psychiatric care, (iv) self-reports. Computerized case records were accessed for each person from all public psychiatric service providers in the Stockholm area since 2007 in making the assessments. Actual psychotic symptoms were rated by an experienced psychiatrist or resident in psychiatry according to the Scale for the assessment of positive symptoms (SAPS) (44)and the Scale for the assessment of negative symptoms (SANS) (45). All were asked about prior interpersonal aggression and drug/alcohol use, and answers recorded.

#### 2.2.2. Healthy individuals

These underwent screening questions in SCAN 2.1 and, when necessary, further questions were asked from the semi-structured interview. Symptom ratings were made on SAPS and SANS. All were asked about prior interpersonal aggression as per the semi-structured interview deployed above and excluded from analyses if rated positive for aggression. Previous and current drug use was recorded and individuals with moderate-high alcohol use confirmed on Peth as well as current drug use were excluded from analyses.

#### 2.2.3. Self-ratings

Self-ratings on AUDIT (Cronbach alpha of 0.80) and DUDIT (Cronbach alpha >0.90) were undertaken by PSD-Agg and healthy individuals. Persons who had scores above clinical cut-off values (females six points on AUDIT, two on DUDIT, males eight on AUDIT, six on DUDIT) were excluded as were those who admitted interpersonal aggression or had current substance abuse as defined by >14 standard alcoholic drinks (10 g alcohol/glass) per week (males) or > 10 drinks for women, or illicit or regular prescribed narcotic intake in the past 3 months.

The Triarchic psychopathy measure (TriPM) with a Cronbach alpha of 0.96 (46, 47) was meant to be rated by all participants, but some individuals with PSD did not agree to doing self-ratings (eight PSD-Agg), four individuals appeared to have missed one page (22 of 58 questions) of the TriPM, and a couple rated the same answer to all questions on the TriPM making the profile invalid. Four made contradictory statements >30% of the time. All these were excluded from the analyses regarding TriPM. Previous research has identified three domains (boldness, meanness, and disinhibition) on previously conducted factor analyses (46). Items are statements about the participant (e.g., “I do not mind if someone I dislike gets hurt”) that are rated on a 4-point Likert scale with response options 0 (false), 1 (mostly false), 2 (mostly true), and 3 (true), with 17 items being reverse coded. Maximum score is thus 174. Items that were left blank within an otherwise valid profile were imputed a value, based on that individual’s average domain score for the missing item’s domain. This left 74 valid profiles in PSD+Agg, 44 in PSD-Agg and 84 in the healthy group.

#### 2.2.4. Investigations

Prior to undertaking the computer presented affect recognition test, the participants were asked to identify the correct synonym for 14 emotion words. There was a choice of three words for each emotion, e.g., for anger – pride, rage, despair; for fear – dread, anger and pleasure. Participants completed the Emotion Recognition Assessment in Multiple modalities (ERAM) task (48). This is a brief emotion recognition test consisting of stimuli from the Geneva Multimodal Emotion Portrayals (GEMEP) corpus (49), a database of actors portraying specific emotions while pronouncing pseudo-linguistic sentences (e.g., “ne kali bam sud molen!”). Pseudowords are used to avoid any potential confounding effects of linguistic content. The ERAM test uses 72 video clips, portraying twelve different emotions (anxiety, despair, disgust, hot anger, interest, irritation, happiness, panic fear, pleasure, pride, relief, and sadness). Each video shows frontal views of the actor’s face and upper torso and provides facial, auditory, and bodily cues of emotion. Ten different actors were shown. The items were presented in three conditions: 24 visual-only items, 24 audio-only items, and 24 audio–visual items, two presentations of each emotion in each condition. The duration of each clip was 1–5 s with sound levels normalized within each of the ten actors. The stimuli were presented within the EQ4A platform (Unity Technologies SF) and after each emotion presentation a list of emotion words appeared on a grey screen, five positive emotions on the left and seven negative emotion words on the right. The twelve emotions were elation/joy, pleasure, interest, pride, relief, anger, irritation, anxiety/worry, panic/fear, despair, sadness, disgust. Presentations were made on a 23” LED-screen (Dell, E2314Hf) in a size of 28 × 20.5 cm. The distance to the test-person was 50–70 cm, dependent on how the person sat. Sound was played on a Dell AY410 Multimedia Speaker System and the volume adjusted for comfortable hearing. The test began with a 24 visual presentations, followed by 24 sound clips and finally the 24 sound and video clips combined. A CSV file of results based on a coding algorithm of which answer was correct was then used to calculate total accuracy for each participant. The task took approximately 20 min to complete.

Psychologist administered matrix reasoning; a subtest of Wechsler Adult Intelligence Scale (50, 51) was used as a measure of fluid reasoning. Age normed scaled scores were obtained from the WAIS manual. A computerized finger tapping test was performed as a measure of psychomotor speed, using an average of number of finger taps per 10 s of the dominant hand. The test was administered using Inquisit 5 software^(^™^)^.^1^

### 2.3. Statistics

Appropriate parametric and non-parametric statistics such as *t* test, Mann–Whitney and Chi-square tests were used to compare groups on demographic data. Spearmans rank correlation coefficient was used in examining relationships between ERAM and TriPM as well as ERAM with symptoms and antipsychotic doses. General linear models were used to probe the relationship between ERAM and variables that may impact ERAM accuracy, starting with all possible determinants and stepwise removing those that were not significant. All analyses were performed in Statistica 14.0.0.

### 2.4. Ethics

All procedures are in accordance with Swedish Research Councils ethical guidelines and the latest Helsinki declarations. Approvals were 2014/827-31/4, 2017/219-32, as well as 2018/307-32/4 and 2019-01422.

## 3. Results

### 3.1. Demographic data

Table 1 shows demographic data for a total of 80 individuals with PSD+Agg, 54 with PSD-Agg and 86 healthy individuals included in this study. As recruitment is ongoing in the PSD-Agg group this group is not well matched for age, they are currently older than the PSD+Agg group and therefore also have a longer illness duration. Typical antipsychotics were more likely to be used in the PSD+Agg group and in higher doses. 35% of PSD+Agg had committed severe acts of aggression resulting in severe bodily injury or death. Over half of participants in the PSD+Agg group have either only completed or not even completed primary school, compared with 14.8% of the PSD-Agg group and just 3.5% in the healthy group. Prior polysubstance or cannabis abuse were much more frequent in the PSD+Agg group than in other groups. WAIS matrix reasoning score was lowest in the PSD+Agg group, intermediate in PSD-Agg and highest in healthy individuals.

**Table 1:** Demographic data.

	PSD+aggression	PSD-aggression	Healthy (c)	Statistics
	*n* = 80 (a)	*n* = 54 (b)	*n* = 86	
Sex - Males	60 (75%)	35 (64.8%)	63 (73.3%)	
Age	34 (20–61)	41.5 (19–57)*	33 (20–60)	8.712, *p* = 0.0128
Education				
Primary school	42 (52.5%)	8 (14.8%)	3 (3.5%)	
Secondary school	29 (36.2%)	34 (63.0%)	31 (36%)	
Vocational college	6 (7.5%)	5 (9.2%)	15 (17.5%)	
University	3 (3.8%)	7 (13.0%)	37 (43%)^‡^	86.89, *p* < 0.0001, 3 groups
Diagnosis				
Schizophrenia	63 (78.8%)	46 (85.2%)	-	
Schizoaffective	0	5 (9.2%)		
Other psychoses	10 (12.5%)	0		
Psychotic Bipolar	7 (8.7%)	3 (5.6%)^‡^		ns
Illness duration	7.5 (0.5–31)	12.0 (0.8–37)^†^	-	2.22, *p* = 0.026, b > a
SAPS	3.0 (0–53)	3.5 (0–79)^†^	0 (0–14)	ns a/b
SANS	23.5 (2–60)	21.0 (0–47)	3 (0–18)^⁑^	ns a/b
Prior Substance use disorder				
Opiates	2 (2.5%)	0	0	
Stimulants	1 (1.25%)	0	0	
Alcohol	8 (10%)	5 (9.3%)	3 (3.5%)	ns
Cannabis	13 (16.25%)	0	1 (1.1%)^‡^	12.22, *p* = 0.0005, a > c
Benzodiazepines	0	2 (3.7%)	0	
Polysubstance use	36 (45%)	9 (16.7%)^‡^	1 (1.1%)^‡^	11.60, *p* = 0.0007, a > b
				43.45, *p* = 0.0001, a > c
				9.80, *p* = 0.0017, b > c
None	20 (25%)	38 (70.3%)	81 (94.3%)	86.87, *p* < 0.0001, c > b > a
Medications				
Typical antipsychotic	38 (47.5%)	8 (14.8%)^‡^	-	15.28, *p* = 0.0001, a > b
Atypical antipsychotic	20 (25%)	26 (48.2%)^‡^		7.66, *p* = 0.0056, b > a
Combination – clozapine+ other	6 (7.5%)	7 (13%)		ns
Atypical and typical antipsychotics				
	16 (20%)	7 (13%)		ns
Lithium	4 (5%)	5 (9,3%)		ns
Other mood stabilizers	12 (15%)	4 (7.4%)		ns
Antidepressants	14 (17.5%)	17 (32%)		ns
Antipsychotic dose	11.75 (0–70)	5 (0–36)^†^	-	3.43, *p* = 0.0006
Most severe aggression		-	-	
Threats to life	12 (15%)			
Assault	40 (50%)			
Grievous bodily harm	25 (31.25%)			
Manslaughter/murder	3 (3.75%)			
WAIS matrix reasoning	7.65 ± 3.26	9.15 ± 4.05^⁑^	11.94 ± 2.89^⁑^	2.31, *p* = 0.022, a < b
				8.85, *p* < 0.0001, a < c
				4.73, *p* < 0.0001, b < c

*Kruskal Wallis, ^‡^Chi^2^, ^†^Mann Whitney U, ^⁑^Students *T* test.

### 3.2. Investigative measures

**Figure 1:**
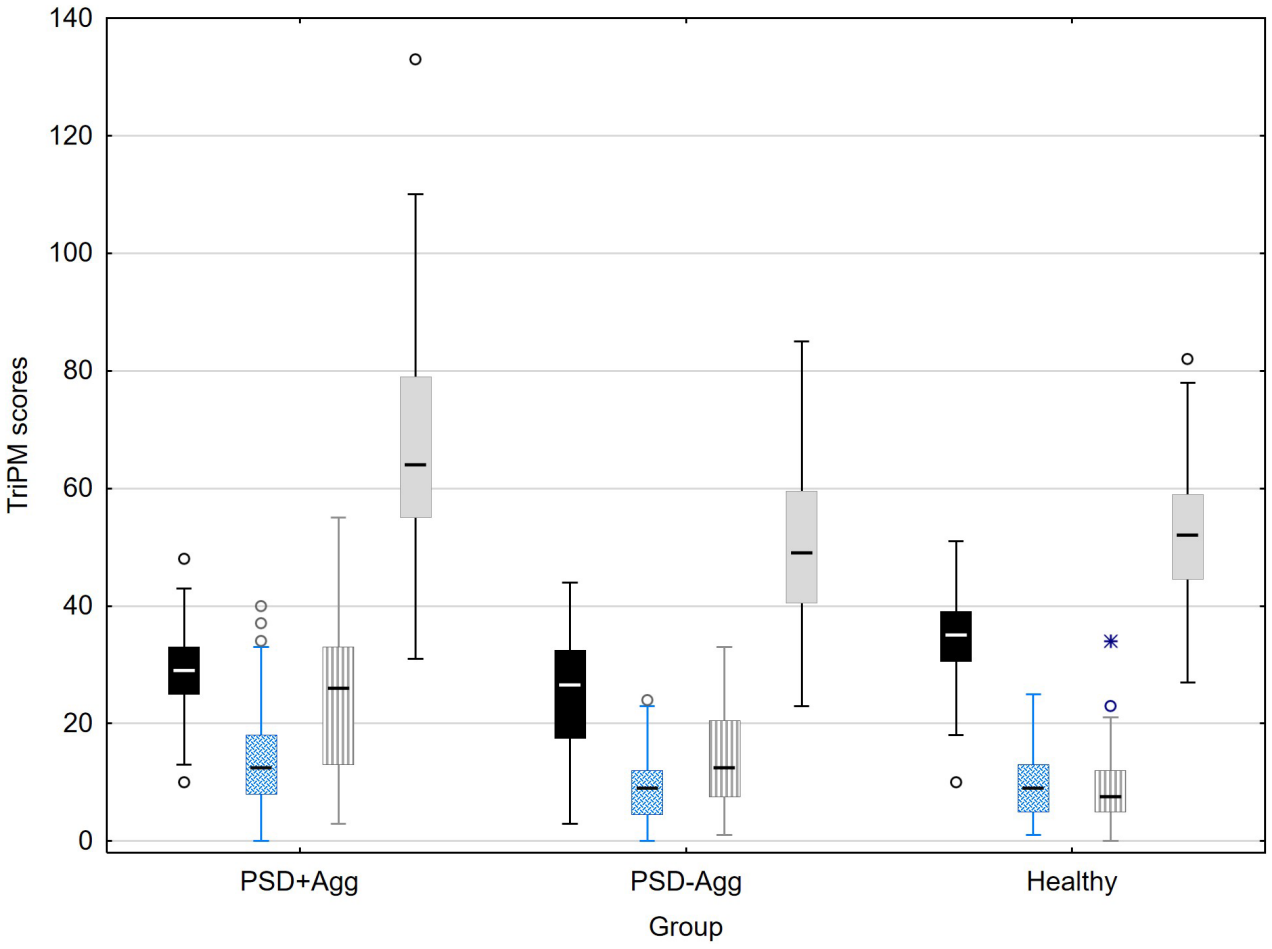
Distribution of TriPM scores in the groups. Median; Box: 25–75%; Whisker: Non-Outlier Range. 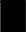 Triarchic psychopathy measure – boldness, 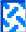 Triarchic Psychopathy measure – meanness, 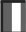 Triarchic psychopathy measure disinhibition, 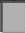 Triarchic Psychopathy Measure total score Outlier and extreme values marked. Statistics see Table 2.

As seen in Figure 1; Table 2, TriPM boldness is lowest in the PSD groups and highest in the healthy group. This contrasts with meanness which is highest in the PSD+Agg group and equally low in the other 2 groups. Disinhibition is by far the highest in the PSD+Agg, intermediate in the PSD-Agg group and lowest in the healthy group. It is this latter difference that most reflects the higher total TriPM score in the PSD+Agg, with no differences between PSD-Agg and healthy groups.

**Table 2:** Group differences in TriPM, finger tapping, and meaning of emotion words.

	PSD+aggression mean (range) (a)	PSD-aggression mean (range) (b)	Healthy mean (range) (c)	Statistics
TriPM^a^				
Boldness	28.6 (10–48)	25.3 (3–44)	39.2 (10–51)	^†^a = b, ns
				a < c, H = 4.58, *p* < 0.00002*
				b < c, H = 5.38, *p* < 0.00001*
				
Meanness	14.2 (0–40)	9.2 (0–24)	9.5 (1–25)	a > b, H = 3.29, *p* = 0.0029*
				a > c, H = 3.53, *p* = 0.0012*
				b = c, ns
				
Disinhibition	24.6 (3–55)	14.4 (1–33)	8.8 (0–34)	a > b, H = 3.75, *p* = 0.0005*
				a > c, H = 8.34, *p* < 0.00001*
				b > c, H = 3.31, *p* = 0.0028*
				
Total score	67.5 (31–133)	48.9 (23–85)	52.5 (27–82)	a > b, H = 5.11, *p* < 0.00001*
				a > c, H = 4.90, *p* < 0.00001*
				b = c, ns
Finger tapping^b^				
Dominant hand	61.0 (39–91)	62.3 (32–97)	72.4 (49–95)	^⁑^a = b, ns
				a < c, *t* = 7.48, *p* < 0.00001*
				b < c, *t* = 5.74, *p* < 0.00001*
Non dominant	53.7 (32–80)	57.4 (30–79)	66.7 (42–95)	^⁑^a = b, ns
				a < c, *t* = 8.21, *p* < 0.00001*
				b < c, *t* = 5.23, *p* < 0.00001*
Correct emotion words ^c^	13 (2–14)	13 (3–14)	13 (2–14)	^†^a = b, a = c, b = c H = 1.39, ns

^a^Based on valid profiles of 74 PSD+aggression, 44 PSD-aggression and 84 healthy, ^b^Taps per 10 second interval on spacebar, ^c^Total of 14 words, median, ^d^Percent correctly identified emotions, ^⁑^Students *t*-test. ^†^Kruskal Wallis. *All significant after correction for multiple testing.

**Figure 2:**
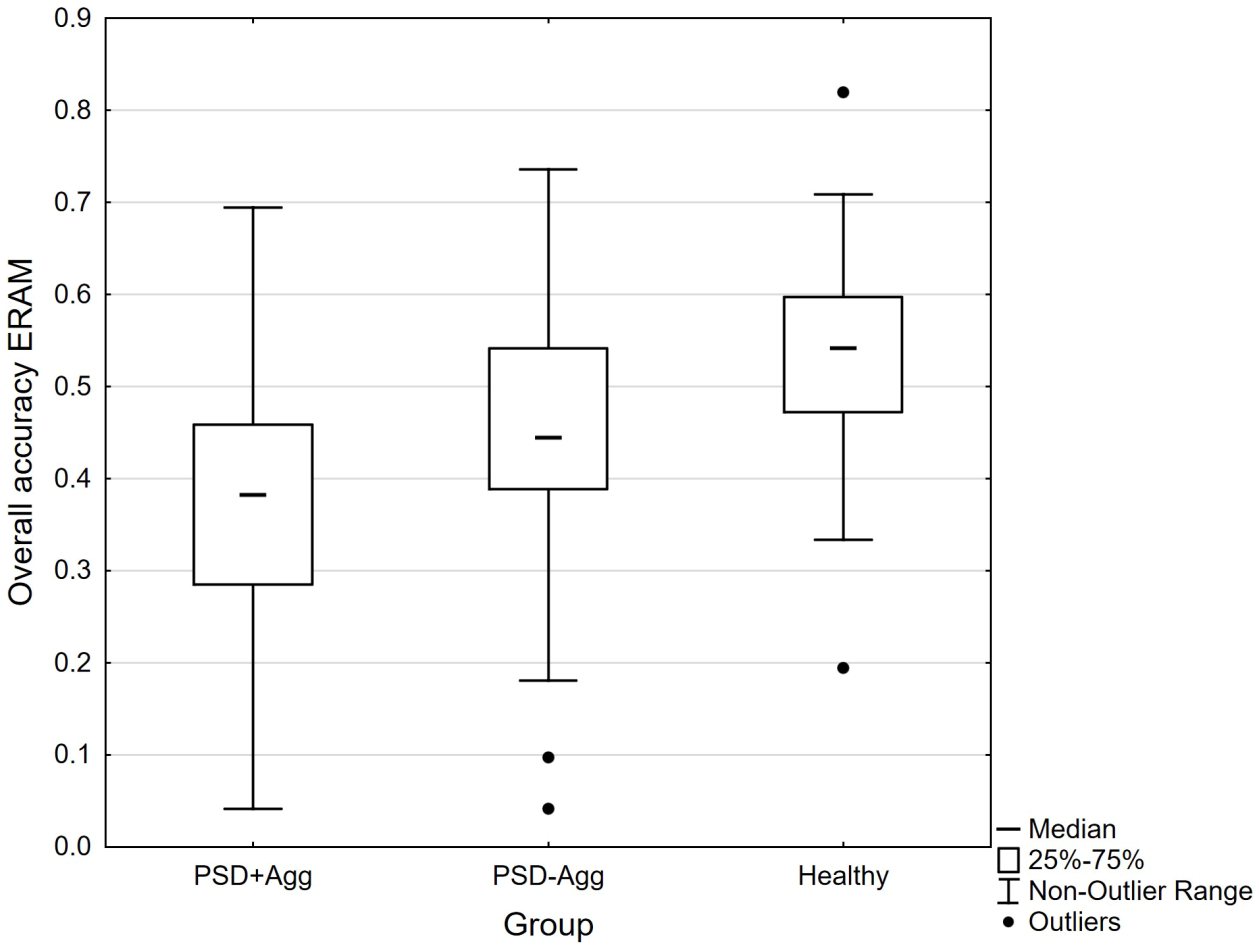
ERAM accuracy Median, 25–75%, Non-Outlier Range, Outliers marked. ERAM: Kruskal Wallis H (2,217) = 57.457, *p* < 0.0001. PSD+Agg (a) mean 36.6 (4.1–69.4), PSD-Agg (b) 44.2 (4.2–73.6), Healthy (c) 53.2 (19.4–81.9). Kruskal Wallis a < b, H = 2.88, *p* = 0.012*, a < c, H = 7.55, *p* < 0.00001*, b < c, H = 3.86, *p* = 0.0003*.

As shown in Table 2, psychomotor speed is equally impaired in both patient groups even though PSD+Agg had higher doses of antipsychotics, and this group were more often prescribed typical antipsychotic agents and combinations of antipsychotic medications. There were no differences between groups on the understanding of emotion words. Figure 2 shows that overall accuracy of emotion recognition in the ERAM test was lowest in PSD+Agg, intermediate in PSD-Agg and highest in healthy individuals.

Table 3 shows correlations between ERAM and TriPM total and subscale scores in the complete sample as well as in each group. There were significant Spearman Rank correlations only at the whole group level. SAPS inversely correlated with ERAM only in the PSD-Agg group (*r* = −0.2694, *p* = 0.04), SANS inversely correlated with ERAM accuracy only in the PSD-Agg group (*r* = −0.4132, *p* = 0.002), while antipsychotic dose inversely correlated with ERAM accuracy only in the PSD+Agg group (*r* = −0.2331, *p* = 0.04). Across patient groups there were no significant relationships.

**Table 3:** Correlations between ERAM and TriPM scores in each group and all individuals.

	Spearman rho	*p* value
All individuals		
TriPM total	−0.1695	0.02
Boldness	0.2132	0.003
Meanness	−0.1411	0.05
Disinhibition	−0.3350	<0.00001
PSD+Agg		
TriPM total	0.0475	ns
Boldness	−0.0023	ns
Meanness	−0.0592	ns
Disinhibition	0.1038	ns
PSD-Agg		
TriPM total	0.076	ns
Boldness	0.0077	ns
Meanness	0.1138	ns
Disinhibition	0.1038	ns
Healthy		
TriPM total	−0.0922	ns
Boldness	0.0912	ns
Meanness	−0.1430	ns
Disinhibition	−0.0922	ns

ERAM, Emotion Recognition Assessment in Multiple modalities; TriPM, Triarchic psychopathy Measure.

Table 4 shows general linear models of determinants of ERAM accuracy across all 3 groups and across PSD groups. There were major effects of WAIS matrix reasoning abilities as well as the understanding of emotion words and psychomotor speed on ERAM accuracy as well as effects of PSD+Agg/PSD-Agg/Healthy group. In order to check if outliers contributed to the highly significant relationship between ERAM and word understanding, the GLM was repeated, excluding the 4 outliers. Outliers were defined as values differing three times the standard deviation of any measure. Word understanding remained highly significant (33.404, *p* < 0.00001 in all three groups and 21.243, *p* < 0.00002 in PSD groups), with group becoming even more important (10.224, *p* < 0.00006 all three groups and 8.313, *p* = 0.0047). There were no significant effects of TriPM total or disinhibition scores in these models. When *post hoc* analyses for power were performed >94% power was obtained when examining all three groups for group, matrix reasoning and word understanding, and over 47% of the variance in ERAM accuracy was explained. Examining just the 2 PSD groups, power was >95% for matrix reasoning and word understanding but dropped to 77% for group effects when just examining PSD groups. In adding TriPM total score, the model explained less of the variance in ERAM accuracy for PSD groups and power for TriPM was 5%. This was also the case for TriPM disinhibition, meanness, or boldness domains (data not shown). Adding SAPS, SANS, and antipsychotic dose to the above model with TriPM, marginally increased the variance explained in the patient groups to 43.3%, up from 42.8%, but power was lost given the large number of variables studied in relation to the sample size. There were no univariate or interactive effects of duration of illness, age or SAPS or SANS nor of antipsychotic dose on ERAM accuracy when taking into account matrix reasoning, word understanding, finger-tapping speed and group. There was 94% power for group in model one (Table 4), 98% power for group in model 5 for all 3 groups and 77% power in model 5 PSD groups only.

**Table 4:** General linear models of determinants of ERAM accuracy.

Model	*F*	*p*	Whole model	*F*	*p*	Whole model
	All 3 groups			PSD groups		
Model 1 All possible factors						
Group (PSD+Agg/pPSD-Agg)				3.055	0.083	
Duration of illness				0.002	0.962	
Antipsychotic dose				0.479	0.490	
SANS				0.118	0.732	
SAPS				0.217	0.642	
TriPM total				0.004	0.951	
WAIS matrix reasoning				7.933	0.006	*F* = 16.227, df 7
Correct synonym				20.655	<0.00002	Adj R^2^ = 0.404
Finger tapping speed				5.692	0.019	*p* < 0.000001
Model 2: All possible factors						
Group	4.273	0.015				
SANS	0.467	0.495				
SAPS	<0.001	0.983				
TriPM total	0.142	0.707				
WAIS matrix reasoning	14.691	<0.0002	*F* = 20.853, df 8			
Correct synonym	31.815	<0.000001	Adj R^2^ = 0.452			
Finger tapping speed	4.273	0.0153	*p* > 0.001			
Model 3: significant variables plus TriPM total						
Group	7.497	<0.0008		4.399	0.038	
TriPM total	0.089	0.765		0.004	0.950	
WAIS matrix reasoning	15.258	0.0001	*F* = 27.952, df 5	8.169	0.005	F = 16.227, df 5
Correct synonym	33.694	<0.000001	Adj R^2^ = 0.457	22.683	0.000006	Adj R^2^ = 0.405
Finger tapping speed	6.393	0.012	*p* < 0.000001	7.379	0.008	*p* < 0.000001
Model 4: significant variables and TriPM disinhibition						
Group	6.868	<0.002		5.973	0.0162	
TriPM disinhibition	0.009	0.922		0.706	0.403	
WAIS matrix reasoning	15.322	<0.001	*F* = 27.92, df 5	8.541	0.004	*F* = 16.474, df 5
Correct synonym	33.709	<0.00001	Adj R^2^ = 0.457	22.600	0.000006	Adj R^2^ = 0.409
Finger tapping speed	6.336	0.013	*p* < 0.000001	7.425	0.008	*p* < 0.000001
Model 5: significant variables only						
Group	10.179	0.00006		7.510	0.007	
WAIS matrix reasoning	21.756	0.000006	*F* = 38.954, df 5	13.868	0.0003	*F* = 24.596, df 4
Correct synonym	41.816	<0.000001	Adj R2 = 0.477	29.732	<0.000001	Adj *R*^2^ = 0.428
Finger tapping speed	4.000	0.047	*p* > 0.000001	4.100	0.045	*p* < 0.000001

## 4. Discussion

In this multimodal emotion portrayal paradigm we found that accuracy of emotion recognition is determined by a diagnosis of psychotic spectrum disorders with the lowest performance occurring in those with a history of aggression. Reasoning ability, the correct understanding of emotion words and psychomotor speed were also important predictors of emotion accuracy. While high self-reported psychopathic traits on TriPM scores were correlated with low accuracy of emotion recognition within the sample as a whole, there was no independent effect of self-rated psychopathy on ERAM when the above factors were taken into account. None of the following predicted ERAM accuracy: positive or negative symptoms of psychosis, duration of illness, or antipsychotic dosages.

Our results are in line with the extensive literature on emotion recognition deficits in schizophrenia (2–4, 9, 20, 22, 37, 52) and in bipolar disorder (2); in video paradigms of emotion portrayals (12), in audio paradigms (15–17, 19) and in point-light display studies (48, 49, 53, 54). Yet our results suggest more severe impairment, especially in those who had been aggressive (31% drop in accuracy for PSD+Agg, and 17% drop for PSD-Agg, compared to healthy individuals), where other studies report a 5–20% drop in accuracy compared with healthy controls. Whether this reflects the greater complexity of the multimodal emotion portrayal test paradigm or is due to the inclusion of individuals with lower reasoning abilities is uncertain. The difference between those with a history of aggression and those without such a history is consistent with other studies (24, 25).

The brain network processing emotion recognition (55) is different to the neurocognitive network (56), and it has thus been assumed that general intellectual capacity will not determine emotion recognition capacity. Yet many studies in schizophrenia have found associations between intellectual ability and measures of social cognition (57–59), even though the relationship does not appear to be a direct one, rather mediated through social reasoning and verbal memory and learning (60). These studies have mostly included individuals with IQ >70, with an average of 90–105, which raises the question if there are more direct associations in those with lower intellectual abilities which we included in this study. Studies in intellectual disability, while fewer and with low sample sizes, suggest increasing impairment with lower IQ (61). These studies have exclusively looked at static faces showing an expressed emotion. Few studies have considered comorbid disorders except for autism, finding no differences controlling for IQ (62). Likewise, there were found to be no differences in emotion recognition between autism and schizophrenia, unless teenagers specifically were examined (63, 64), alluding back to the earlier suggestion that emotion recognition is less severe in the prodromal, early phase of the schizophrenia (10). Thus, affect recognition is impaired in a range of conditions where impairments in intellectual ability is one strong predictor of poorer performance, perhaps explaining why the schizophrenia polygenic risk score did not *per se* give lower social cognition and emotion recognition scores (8).

Many of the studies conducted with individuals with schizophrenia did not check for semantic understanding of emotion words prior to testing; when done it led to the exclusion of those subjects who did not know the meaning of the word from the test. This may tease apart emotion recognition difficulties from poor understanding of the emotion word label, yet we do not know how many individuals suffering from schizophrenia have semantic difficulties in naming and understanding emotion words which may underlie problems recognizing others emotional states. All existing tests of emotion recognition rely not only on a visual/auditory presentation of emotion in a person, but they also all include a presentation of emotion words for the subject to choose from. Individuals who may not know the precise meaning of an emotion word can have previously coded it as an emotional valence, showing reduced accuracy of word understanding but still appreciating whether it is a positive or a negative feeling state. While we think of emotion and valence of emotion as being similar concepts, a meta-analysis of imaging studies has shown different neural substrates. When emotion words (e.g., “anger” and “disgust”) were presented, regions related to semantic processing were activated, as opposed to the amygdala and parahippocampal gyrus being activated when valence words such as “pleasant” and “unpleasant” were presented (65). While this suggests that in test paradigms we tap into semantic and cognitive networks as well as the network of emotion recognition, it is also possible that the lack of a vocabulary for specific “unpleasant” emotions leads to arousal and autonomic dysregulation, potentially leading to an aggressive response. While there were no differences between the groups on understanding synonyms to our emotion words, understanding what an emotion word means remained a powerful predictor of emotion recognition accuracy and was significant in all the general linear models. It would be of great interest to delve more into the semantic confusion of word meanings, alexithymia and their relationships with emotion recognition and aggression.

There were negative correlations between self-rated psychopathic traits with emotion recognition accuracy within the whole group of 202 subjects who had a valid TriPM profile. Yet within each subgroup there were no correlations, and when considering reasoning ability, psychomotor speed and understanding of emotion words there was no independent effect of TriPM scores on emotion accuracy scores, confirming the study by Dawel et al. (33). This does not preclude an effect of psychopathic traits on emotion recognition given that a disproportionate number of individuals in the PSD-Agg did not validly complete the TriPM self-rating (10/54, 18.5%) along with 6/80 (7.5%) of the PSD+Agg. In fact, our findings conflict with (30) as well as population samples with higher psychopathy ratings which have shown impairment in emotion recognition (33). However, these studies did not consider the effect of reasoning ability and psychomotor speed on their results. While we were unable to find associations between self-rated psychopathic traits and emotion recognition deficits this does not preclude that individuals with higher degrees of psychopathic traits have difficulties in emotion recognition. Less than 25% of individuals in our group of PSD+Agg scored above 80 on the TriPM, above which no healthy individuals or PSD-Agg individuals scored in this study. While we also found no associations with disinhibition, which largely accounted for the difference in total TriPM scores, a link between aggression and impulsiveness is well documented in non-psychotic populations (66) the type of aggression varying with personality subtype (67, 68).

Limitations of our methodology include a cross-sectional design, limiting inferences of causality, and our reliance on self-reported psychopathic traits. We did not have access to more rigorous interview and observation-based measures of psychopathic behaviors or traits. The reliance on self-ratings may have lowered diagnostic accuracy and prevented us from seeing effects on emotion recognition. A greater number of absent and invalid scoring profiles in the PSD-Agg group were make it also possible that this underestimates psychopathic traits in this group, and potentially in the PSD+Agg group where we had invalid TriPM ratings. On the other hand, it may be that the sample did not exhibit sufficiently high psychopathy ratings thus being unable to differentiate the effects of psychopathy. Other limitations of our study involve the use of ERAM which employs only Caucasians with an obvious French accent, which may affect how non-Caucasians interpret emotion portrayals (61, 62). Additionally, the presentation clips are between one and five seconds which may negatively affect the ratings by those who have slow psychomotor processing speed in the PSD groups, artificially reducing their performance. As we analyzed accuracy based on all types of presentations of emotions, we are unable to distinguish if one or other modality is more affected in the PSD groups. Neither have we analyzed if strong or weak intensity of emotions yield differences with respect to psychopathy. We are planning to examine these contributions in future work. While we had power for analyzing the contributions of reasoning, psychomotor speed, and group contributions, the sample number was too low to fully address the effect of psychopathic traits, a reanalysis of this will be conducted when the full sample of PSD-Agg has been obtained.

A strength of our study is the use of a validated multimodal emotion portrayal paradigm using body posture, facial expression and voice. This is judged to be more ecologically valid than the usual Ekman faces in examining emotion recognition and allows for a greater range of emotions, especially of positive feeling states and of differing intensities. Furthermore the inclusion of patients who have comorbidities in terms of prior substance abuse and intellectual challenges, creates more ecologically valid and generalizable findings to the group of persons with psychoses who commit aggressive acts, mostly during psychotic episodes. The Swedish system of extended inpatient forensic psychiatric care enabled such individuals to be in remission from their substance use disorders, which is otherwise a source of confounding in studies of individuals with psychosis and aggression. Another strength is that interviewers, testers, and symptom raters were blind to the ERAM results. Additionally, variation in diagnosis or symptom ratings were minimized by having only two raters of aggression and three raters of symptom and diagnoses, with consensus ratings of aggression, diagnoses and symptoms. A variety of sources of information also contributed to a high reliability of the clinical information.

## 5. Conclusion

We failed to identify an independent relationship between self-rated psychopathic traits and multimodal emotion recognition in individuals with psychotic spectrum disorders with respect to a history of aggression. A history of aggression, patient status, reasoning, psychomotor speed and understanding of emotion words accounted for 47% of the variance in emotion recognition scores on ERAM. Future studies will examine if self-rated psychopathy and/or a history of aggression impact the recognition of specific emotions and whether emotions with positive or negative valence are differentially impacted.

## Data availability statement

The raw data supporting the conclusions of this article will be made available by the authors, without undue reservation.

## Ethics statement

The studies involving human participants were reviewed and Approvals by Stockholm Ethics board 2014/827-31/4, 2017/219-32, as well as 2018/307-32/4 and by Swedish Ethical Review Authority 2019-01422. The patients/participants provided their written informed consent to participate in this study. Written informed consent was obtained from the individual(s) for the publication of any potentially identifiable images or data included in this article.

## Author contributions

LH, HF, MKr, and AJ were instrumental in designing the study. AJ wrote the grant applications to enable the study to be performed. PL developed ERAM. AJ trained test-leaders to administer ERAM. Testing was conducted primarily by GG and MKä. AJ undertook diagnostic and symptom ratings. AJ and LH undertook the statistical analysis. LH and AJ wrote the first draft of the manuscript. All authors reviewed and accepted the final manuscript.

## Funding

Funding was provided by Vetenskapsrådet (2021-06362), Region Stockholm (ALF-952824 and 951046), Svenska Läkaresällskapet (779731 and 589661), and Fredrik and Ingrid Thuring’s fund (00127 och 00264).

## Conflict of interest

The authors declare that the research was conducted in the absence of any commercial or financial relationships that could be construed as a potential conflict of interest.

## Publisher’s note

All claims expressed in this article are solely those of the authors and do not necessarily represent those of their affiliated organizations, or those of the publisher, the editors and the reviewers. Any product that may be evaluated in this article, or claim that may be made by its manufacturer, is not guaranteed or endorsed by the publisher.

## Footnotes

^1^ https://www.millisecond.com
